# Viral Sepsis: Pathogenesis, Diagnostics and Therapeutics—A Summary of Key Findings and Insights from the Special Issue

**DOI:** 10.3390/v17121601

**Published:** 2025-12-10

**Authors:** Stamatia Tsoupra, Karolina Akinosoglou

**Affiliations:** 1 Department of Medicine, University of Patras, 26504 Rio, Greece; stamtsoupra@gmail.com; 2 Department of Internal Medicine, University General Hospital of Patras, 26504 Rio, Greece; 3 Division of Infectious Diseases, University General Hospital of Patras, 26504 Rio, Greece

Viral sepsis has gained increasing clinical and scientific attention as it represents a complex, life-threatening condition driven by a dysregulated host response to viral pathogens. In this Special Issue, “Viral Sepsis: Pathogenesis, Diagnostics and Therapeutics,” eight contributions collectively deepen our understanding of the biological mechanisms, immunological manifestations, diagnostic clinical challenges, and therapeutic avenues related to viral sepsis.

A main issue across several studies concerns the complex pathogenesis of COVID-19 sepsis-like syndrome and the immune response of the host to infection, characterized by systemic inflammation, endothelial dysfunction and coagulation abnormalities. Two prospective cohort studies from Eastern Europe highlight the prognostic significance of inflammatory biomarkers and radiological findings. Mateescu et al. identify D-dimer, CRP, and >50% lung involvement on CT scan as independent predictors of poor outcomes in COVID-19 sepsis, underscoring the interconnected roles of inflammation and coagulation in disease severity [1]. Complementing these findings, Mateescu and colleagues further demonstrate that elevated IL-6, troponin, NT-proBNP, severe lung involvement, unvaccinated status, and higher BMI independently predict adverse outcomes, while exploratory data suggest a possible survival benefit from tocilizumab therapy [2].

A third study adds nuance to the biomarker landscape by evaluating presepsin in COVID-19 [3]. While levels were highest in severe disease and correlated with procalcitonin, the authors found that this rise was largely driven by HSV-1 reactivation rather than bacterial or fungal superinfection [3]. Together, these three studies highlight the complexity of interpreting inflammatory markers in viral sepsis and reinforce the need for integrated biomarker-based risk stratification, accounting for latent viral reactivation when assessing disease severity.

Beyond COVID-19, this Special Issue expands the perspective on viral sepsis to include syndromes characterized by hyperinflammation and immune dysregulation. Two reviews by Papageorgiou et al. and Livieratos et al., respectively, highlight the dual nature of immune responses in viral infections [4,5]. The first review details how macrophage activation syndrome (MAS) represents the extreme end of immune dysregulation, with viral pathogens such as SARS-CoV-2, EBV and influenza driving cytokine storm and severe hyperinflammation [4]. In contrast, the second review focuses on the adaptive side of immunity, examining how vaccination, viral variants, and hybrid immunity shape inflammatory profiles and long-term COVID-19 outcomes [5]. Viewed together, these articles highlight the delicate balance between protective immunity and pathological inflammation that defines the clinical course of viral sepsis and underscore the need for validated diagnostic criteria and targeted immunomodulatory strategies.

Improving diagnostic differentiation between viral and bacterial sepsis remains a persistent clinical need. In a prospective, two-center observational study, Perschinka et al. highlighted soluble Neuropilin-1 (sNRP-1) as a promising biomarker for distinguishing bacterial from viral sepsis. While traditional markers such as IL-6, CRP, and procalcitonin declined rapidly over time, sNRP-1 remained consistently elevated in bacterial sepsis, offering clearer diagnostic separation [6]. These findings demonstrate that sNRP-1 may serve as a potential tool to differentiate between bacterial and viral pathogens in sepsis.

Two studies in this Special Issue emphasize how viral sepsis manifests differently across vulnerable populations [7,8]. The first examines pregnant and postpartum women admitted to the ICU with severe COVID-19, showing that although pregnancy was associated with higher rates of ARDS and distinct biochemical profiles, such as lower ferritin, lactate, and LDH, outcomes were ultimately comparable to age-matched nonpregnant women [7]. In contrast, the analysis of Roma patients in Western Romania underscores how social disadvantage amplifies clinical risk, with this group exhibiting more frequent ICU admissions, more severe symptomatology and higher inflammatory biomarker levels, including markedly elevated CRP and IL-6 [8]. Together, these studies illustrate how both physiological vulnerability and social marginalization shape the clinical pattern and severity of viral infections, reinforcing the need for personalized medical care approaches in diverse patient groups.

Beyond the COVID-19-focused contributions, viral sepsis represents a broader and increasingly recognized clinical entity encompassing pathogens such as influenza, RSV, enteroviruses, adenoviruses, herpesviruses, hantaviruses, and viral hemorrhagic fevers. These infections can trigger profound immune dysregulation, endothelial injury, and multiorgan failure, yet their mechanisms remain incompletely defined. Key unmet needs include improving early distinction between viral and bacterial sepsis, identifying pathogen- and host-specific biomarkers, understanding the role of viral persistence and reactivation, and developing targeted immunomodulatory therapies. Future research will be crucial for refining diagnostics and guiding personalized therapeutic strategies in viral sepsis.

In conclusion, the contributions to this Special Issue offer a multidimensional view of viral sepsis, from molecular mechanisms and diagnostic innovation to vulnerable populations and therapeutic prospects. As viral pathogens continue to challenge healthcare systems globally, advancing precise diagnostics, targeted immunomodulation and personalized medical treatment remain essential priorities. We hope this collection will inspire further research and support clinicians and scientists working to improve outcomes in viral sepsis.
